# Message Appeals on an Instagram Account Promoting Seat Belt Use That Attract Adolescents and Young Adults: Elaboration-Likelihood Perspective Study

**DOI:** 10.2196/16800

**Published:** 2020-09-28

**Authors:** Ni Zhang, Stacy A Drake, Kele Ding

**Affiliations:** 1 San Jose State University San Jose, CA United States; 2 Department of Industrial and Systems Engineering Texas A&M University College Station, TX United States; 3 Kent State University Kent City, OH United States

**Keywords:** rational appeal, ego appeal, social appeal, fun appeal, positive emotional appeal, fear appeal, social media, youth, adolescents, health information, car safety

## Abstract

**Background:**

Adolescents and young adults demonstrate the highest rate of unrestrained motor vehicle fatalities, making the promotion of seat belt restraint a priority for public health practitioners. Because social media use among adolescents and young adults has proliferated in recent years, it is critical to explore how to use this tool to promote seat belt use among this population. Social media posts can contain various types of information within each post and this information can be communicated using different modalities.

**Objective:**

In this study, based on the elaboration likelihood model, we aimed to examine how adolescents and young adults reacted to different appeals in various components of posts in the pilot of a promotion intervention on the Instagram *BuckleUp4Life* account.

**Methods:**

Using thematic analysis, we examined different appeals in 3 components (photo, text, and caption) of 199 posts in *BuckleUp4Life* and compared the number of likes for different appeals.

**Results:**

We found that 6 appeals were used in the posts: rational, ego, social, fun, positive emotional, and fear appeals. The results of our study showed that in photos, fun appeals were the most popular. Rational and positive emotional appeals were the most appealing in text and captions. Regardless of the location of the components (photo, text, or captions), rational appeal was the most popular appeal.

**Conclusions:**

Based on the findings of our study, we recommend that public health practitioners utilize fun photos with rational and positive emotional appeals in text and captions rather than fear or social appeals, when promoting seat belt use through social media, especially Instagram.

## Introduction

### Background

Despite improvements in vehicle safety, motor vehicle crashes remain a major public health concern and a leading cause of death in the United States (Centers for Disease Control and Prevention) [1]. While there has been an overall increase in seat belt restraint use, accompanied by a decrease in unrestrained fatalities, 49% of motor vehicle fatalities involve unrestrained occupants (National Center for Statistics and Analysis) [2]. Additionally, adolescents and young adults represent the largest proportion of unrestrained fatalities [2]. As motor vehicle crashes and fatalities continue to be a significant health concern among adolescents and young adults, social media platforms may provide an effective means to promote motor vehicle safety (National Highway Traffic Safety Administration) [3].

Most teenagers in the United States between the ages of 13 years and 17 years report using social media sites such as YouTube (85%), Instagram (72%), Snapchat (69%), Facebook (32%), and Twitter (32%) [4]. These levels of social media use among teenagers have led health organizations and health care professionals to recognize that social media sites are viable platforms for public health communication and for adopting new communication channels for health promotion interventions [5,6]. Several research studies have shown how professionals have used social media, particularly Facebook [7] and Twitter [8], for health communication in different public health fields. For example, researchers have examined how social media sites have been used to promote physical activity [8-10], diabetes awareness, and mental health [11]. However, no researchers, to our knowledge, have examined how social media sites are or could be used as a health intervention to promote seat belt use.

While social media posts can potentially be used to promote seat belt use among adolescents and young adults, it is still unclear what kinds of persuasive appeals in this new medium are most effective at attracting the young generation. Previous research has examined the appeals of mass media [12] and websites [13] in road safety communication campaigns. Guttman [13] examined persuasive appeals in over 300 road safety health communication campaigns from 41 countries, mainly on websites of road safety organizations. Guttman categorized 5 main types of persuasive appeals, namely, appealing to reason, appealing to “negative” and “positive” emotions, the threat of enforcement, and the use of humor.

According to the elaboration likelihood model (ELM), there are 2 routes by which outside messages influence and persuade an individual: the central and peripheral routes of persuasion [14]. The central route describes the perception of a message that health educators think about (ie, elaborate upon) more thoroughly compared to the peripheral route, in which less thought occurs at a lower elaboration level. Rational appeal, one of Guttman’s categories of persuasive appeals, requires the audience to go through the central route, which focuses on the content and arguments of the message [13]. The ELM asserts that people are more likely to process information through a central route when the information requires reasoning or thorough understanding [14]. The ELM shows that people can be attracted to peripheral cues or appeals such as liking, consensus, or credibility, rather than the content or the argument of the message [14]. The “negative” and “positive” emotions, the threat of enforcement, and the use of humor in Guttman’s categorization can peripherally persuade an audience [13]. For example, the National Cancer Institute reported in as early as 2004 that positive emotional appeals were better accepted when viewers comprehended the action portrayed in the message and that health communication campaigns should avoid high degrees of fear arousal, unless the fear is easily resolvable [15]. In addition, the use of humor could attract an audience to a particular content because enjoyment is one of the main reasons people use social networking sites [16].

According to Witte et al [17], people consider fear as either a motivator or an inhibitor for behavioral change depending on their perceptions of the risks and their willingness to change the situation or their behaviors. Some have recommended that health communication campaigns avoid high degrees of fear arousal unless the fear is easily resolvable [15]. However, very few research studies have examined different appeals about seat belt use promotion on Instagram. Furthermore, only few studies have explored the appeals that attract the young audience on Instagram.

Of the main social media sites, Instagram is one platform in which one can use both the central and peripheral routes of persuasion [18]. Instagram is a web-based social networking site that allows users to share photos and videos with texts directly. On Instagram, users can post images or videos permanently on their profile or as stories, which disappear from users’ profiles, feeds, and direct inboxes after 24 hours. Instagram posts can serve to inform, entertain, or express an opinion [18]. Appeals in both the central and peripheral routes can be presented in one or more of these 3 components of a post: photo, text, and caption. Additionally, Instagram users may publicly display their reactions to other user’s content by “liking” a post or commenting on a post.

The *BuckleUp4Life* account on Instagram was created for a pilot Instagram intervention promoting the use of seat belts among high school adolescents and young adults [19]. It was developed in the fall of 2014 with approximately 1000 followers, and this account included high school adolescents and young adults by the end of 2015. This account was open to the public with a hashtag #SeatbeltSelfie. This site was promoted by a group of 20 undergraduate nursing students who inputted 1-3 daily posts that resulted in an average of 19 likes per day in the *BuckleUp4Life* Instagram account. Working with the injury prevention program in the Level 1 trauma department of a local hospital, the nursing students used 3 high school health fairs to advertise the *BuckleUp4Life* Instagram account. The high school adolescents and nursing students could also invite their friends to follow the account. Thus, the followers of this account were not limited to high school adolescents and undergraduate nursing students. This pilot health communication intervention provided an opportunity to observe the persuasive appeals that the nursing students used to collect data on the audience reactions to the different appeals about seat belt use promotion.

### Research Questions

Our research examined the types of persuasive appeals used by the *BuckleUp4Life* Instagram account and how those appeals were received by the audience.

Our first research question (RQ1) was as follows: What are the different types of persuasive appeals in different components (photo, text, and caption) of the posts in the *BuckleUp4Life* Instagram account?

Our second question (RQ2) was as follows: Are the distributions of the types of appeals different among different components (photo, text, and caption) in the posts in the *BuckleUp4Life* Instagram account?

Our third research question (RQ3) was as follows: What is the relationship between the different appeals and the number of likes in the different components of the post (photo, caption, and text)?

The fourth research question (RQ4) was as follows: What are the differences in the number of likes among the different appeals, regardless of the components of the posts?

## Methods

We used thematic analysis [20] to code the appeals that appeared in each component (photo, text, and caption) of 199 posts occurring between September 2014 and September 2015 in the *BuckleUp4Life* Instagram account [21]. Thematic analysis was used to pinpoint, examine, and record patterns of meaning (or “theme”) within each post. Additionally, we recorded the number of likes for each appeal in each component of the posts on Instagram.

To ensure rater accuracy and coding reliability, 2 public health students used 5 rounds of coding with 49 posts to obtain a consensus on the component’s appeal categories. The first author met with the 2 raters to discuss the disagreement and reconcile them in each round of coding and revised the coding scheme accordingly. To test interrater reliability, we independently coded 49 posts by indicating “yes” or “no” to each appeal identified on 3 aspects of a post: photo, text, and caption. Holsti tests indicated an interrater reliability of 77%-95% for the 5 rounds of coding. After the interrater reliability reached a satisfactory level (>0.80) for each appeal, 2 research principals coded the 199 posts.

We used descriptive statistics to examine the distribution of the number of likes each appeal received for the 3 post components (photo, text, and caption; RQ2). Because the number of likes for each characteristic were not normally distributed, we used Mann-Whitney *U* tests to compare the mean number of likes for each element within the same component to the mean number of likes of all the remaining cases (RQ3). We examined the similarity of the histogram distribution of any 2 pairs of elements entering a test prior to the Mann-Whitney *U* test. We aggregated the same characteristic across 3 aspects, examined the normality and outliers for the number of likes, and compared all the characteristics for their mean number of likes by using one-way analysis of variance (RQ4). We set the alpha at .05 and used SPSS 25 (IBM Corp) for all statistical analyses.

## Results

### Analysis of RQ1 and RQ2

We identified 6 main appeals used in the different components of the posts in the *BuckleUp4Life* Instagram account: rational, ego, social, fun, positive emotional, and fear (Table 1).

Rational appeal: We identified the rational appeal as using logical reasoning or the central route of persuasion to provide justification for seat belt use. We found 4 rational appeals in photos, 38 in text, and 52 in captions.

Ego appeal: Instagram users can post selfies while wearing seat belts and share their own seat belt wearing experience. We identified ego appeal as selfies (self/group images taken by an individual in the photo) and any information bolstering of self-image of wearing a seat belt. We found 55 selfie appeals in photos, 8 in texts, and 9 in captions.

Positive emotional appeal: We identified positive emotional appeals as showing or promising positive emotions, promise of acceptance, love, or related rewards. We found 10 positive emotional appeals in photos, 26 in text, and 27 in captions.

Fun appeal: We included humor, sarcasm, jokes, excitement, and entertainment as fun appeals. For example, we identified cartoon characters and toys as fun appeals primarily because of their entertainment characteristics. We found 42 fun appeals in photos, 18 in texts, and 6 in captions.

Fear appeal: We identified fear appeal as any information that could evoke fear. We identified 8 fear appeals in photos, 12 in texts, and 9 in captions.

Social appeal: We defined any collective actions, including social events and gatherings for seat belt use promotion, as social appeals. Appeals to social value cater to solidarity or collective actions. Collective actions, including social events, demonstrate social support in an offline setting. We found 34 social appeals in photos, 17 in texts, and 20 in captions.

**Table 1:** Association between the number of likes and appeals in different components of the Instagram posts (N=199).

Components, appeals, and likes			n (%), Values	Mean	*U* (Mann-Whitney)	*P* value
**Photo**						
	**Fun**				1641	.001
		Yes	42 (21.1)	19		
		No	157 (78.9)	35		
	**Fear**				668.5	.56
		Yes	8 (4.1)	23		
		No	191 (95.9)	32		
	**Rational**				177	.06
		Yes	4 (2.1)	59		
		No	195 (97.9)	31		
	**Social**				646.5	.001
		Yes	34 (17.1)	13		
		No	165 (82.9)	35		
	**Positive emotions**				913	.88
		Yes	10 (5.1)	24		
		No	189 (94.9)	32		
	**Ego**				3236.5	.05
		Yes	55 (27.6)	25		
		No	144 (72.4)	34		
	**None**				2261.5	.001
		Yes	80 (40.2)	43		
		No	119 (59.8)	23		
**Text**						
	**Fun**				1507.5	.63
		Yes	18 (9.1)	39		
		No	181 (90.9)	30		
	**Fear**				840	.15
		Yes	12 (6.1)	34		
		No	187 (93.9)	31		
	**Rational**				1965.5	.001
		Yes	38 (19.1)	40		
		No	161 (80.9)	29		
	**Social**				536	.001
		Yes	17 (8.5)	14		
		No	182 (91.5)	33		
	**Positive emotions**				1425	.003
		Yes	26 (13.1)	41		
		No	173 (86.9)	30		
	**Ego**				657	.52
		Yes	8 (4.1)	21		
		No	191 (95.9)	32		
	**None**				3966.5	.02
		Yes	92 (46.2)	27		
		No	107 (53.8)	35		
**Caption**						
	**Fun**				366.5	.13
		Yes	6 (3.1)	43		
		No	193 (96.9)	31		
	**Fear**				665	.27
		Yes	9 (4.5)	31		
		No	190 (95.5)	31		
	**Rational**				2596.5	.001
		Yes	52 (26.1)	38		
		No	147 (73.9)	29		
	**Social**				857.5	.001
		Yes	20 (10.1)	16		
		No	179 (89.9)	33		
	**Positive emotions**				1495.5	.003
		Yes	27 (13.6)	39		
		No	172 (86.4)	30		
	**Ego**				604	.14
		Yes	9 (4.5)	18		
		No	190 (95.5)	32		
	**None**				3760	.009
		Yes	85 (42.7)	29		
		No	114 (57.3)	33		

### Analysis of RQ3

People liked some *BuckleUp4Life* posts much more than the others, with the number of likes per post ranging from 7 to 158. We found that for appeals in photos, fun demonstrated a significantly positive association with the number of likes (Table 1, *P*<.001). For appeals in text and in captions, rational and positive emotional appeals had significantly positive associations with the number of likes (*P*<.001). Social appeal had a significantly negative association with the number of likes for photos and captions (*P*<.001) (Table 1).

### Analysis of RQ4

To examine the differences in the number of likes among the different appeals regardless of their locations in different components of the posts (RQ4), we averaged the number of likes for each appeal across the 3 components (photo, text, and caption). We found a significant difference in the number of likes across the 6 appeals (F=8.537, *P*=.01). Tukey’s honest significant difference postdoc testing revealed that the number of likes for social appeal was significantly lower than that for fear, ego, positive emotional, and rational appeals (*P*=.04, *P*=.02, *P*=.01, and *P*=.01, respectively). The number of likes for fun was significantly lower than that for positive emotional and rational appeals (*P*=.02). The number of likes for fear was significantly lower than that for rational appeal (*P*=.05). The number of likes for ego appeals was significantly lower than that for positive emotional and rational appeals (*P*=.01, Table 2).

**Table 2:** Comparison of the mean number of likes among the different appeals in Instagram posts.

Type of appeal (mean number of likes)		Social (4.9)	Fun (8.1)	Fear (9.1)	Ego (10.7)	Positive emotional (12.9)	Rational (15.4)
**Social (4.9)**							
	d^a^	0	3.2	4.2	5.8	8.0	10.5
	*P* value^b^	—^c^	.16	.04	.02	.001	.001
**Fun (8.1)**							
	d	—	0	1.0	2.6	4.8	7.3
	*P* value	—	—	.89	>.99	.051	.02
**Fear (9.1)**							
	d	—	—	0	1.6	3.8	6.3
	*P* value	—	—	—	.81	.82	.051
**Ego (10.7)**							
	d	—	—	—	0	2.2	4.7
	*P* value	—	—	—	—	.03	.006
**Positive emotional (12.9)**							
	d	—	—	—	—	0	2.5
	*P* value	—	—	—	—	—	>.99
**Rational (15.4)**							
	d	—	—	—	—	—	0
	*P* value	—	—	—	—	—	—

^a^One-way analysis of variance test was performed. “d” values are the differences between the mean number of likes among the different appeals.

^b^*P* values were obtained from Tukey posthoc test.

^c^Not applicable.

## Discussion

### Principal Results

The posts on *BuckleUp4Life* Instagram account used 6 main appeals within 3 components of each post (photo, text, and caption): rational, ego, social, fun, positive emotional, and fear. Most of these appeals were consistent with Guttman’s identification of 5 main appeals found mostly on the websites of road safety organizations [13]. Specifically, the rational appeal identified in this study is similar to Guttman’s appealing to reason. Similarly, the positive emotional appeal identified in this study is consistent with Guttman’s categorization of positive emotional appeals with regard to what people were concerned about including compassion, empathy, and caring [13]. Moreover, the fun appeal identified in this study is similar to Guttman’s use of humor appeal, but our study broadened this category to include sarcasm, jokes, excitement, and entertainment [13]. The fear appeal identified in our study is similar to Guttman’s threat of enforcement [13].

We identified 2 appeals in the *BuckleUp4Life* Instagram account that were not similarly identified in Guttman: ego and social appeals. The ego appeal is unique to Instagram because this social media platform is user-generated and thus different from traditional mass media [13]. Users can post their own photos and report their own seat belt wearing status. Social appeal may also be unique to social media, and thus not identified in Guttman’s study because people use social networking sites to connect with each other for social purposes [13]. Furthermore, social networking sites have been found to provide social support for other health behaviors such as physical activity [10].

There was a wide range in the number of likes per post (7-158), suggesting that in viewing and responding to posts, the audience as a group either greatly liked or did not like a post. Additionally, the *BuckleUp4Life* Instagram program demonstrated that high school students preferred rational appeals over fun, fear, social, positive emotional, and ego appeals, regardless of whether the appeal was imbedded within a photo, text, or caption. In particular, rational appeals presented within the text, either through captions or superimposed on a photo, were more likely to yield likes than those posts that did not have a rational appeal. Because the *BuckleUp4Life* Instagram audience was high school adolescents and young adults, this young population may need more information to make decisions [14]. Thus, rational appeal was more attractive to our young audience as they went through the central route of information processing when viewing a post.

Peripheral route appeals also attracted the *BuckleUp4Life* Instagram audience. Positive emotional appeals in text or captions received significantly more likes than post components that did not have positive emotional appeals. Our findings may be due to students’ positive emotions toward seat belt use in terms of their acceptance of the behavior. Our study confirmed that positive emotional appeals are attractive to adolescents and young adults in terms of seat belt use promotion. Positive emotional appeals received more likes than fun appeals, regardless of the location in an Instagram post. Fun appeals were more likely to receive likes in photos than in texts and in captions. Furthermore, our findings suggest that fun appeal is easily achievable through pictures that do not directly relate to people wearing seat belts. For example, a picture of a stuffed bear in a car wearing a seat belt received many likes. Fun appeal uses humor to entertain and helps viewers overcome their reluctance in viewing safety messages.

In contrast, the peripheral route ego appeal was not popular among the audience, as people preferred fun photos to selfies depicting individuals wearing seat belts. This could be because the posts were not generated by people that the audience knew. Ego appeal might be more effective within their own Instagram networks rather than on a public Instagram account such as *BuckleUp4Life*.

Social appeal was the least popular of the 8 appeals identified in this study. Social gatherings and events to promote seat belt use might not be of interest to this population, as individuals in this young population may consider seat belt use a personal decision that is easy and that does not require social support in the way that other health behaviors such as physical activity require [10]. Social appeal might not have influenced likes because the audience may not know the people who posted the information about a particular social gathering and thus, they felt less engaged with the social appeals.

We found that our audience did not like fear appeals. Witte et al [17] suggested that young people may not perceive the risks involved in not using a seat belt and thus, may fail to see fear as a motivator to change their behavior. Therefore, our finding that fear appeals were not significantly associated with likes may be due to the age range of our subjects.

### Applications

The goal of this study was to investigate ways in which health educators can use Instagram as an effective health communication channel. This study demonstrated that rational appeals were more effective than other appeals in garnering the positive attention of high school students when promoting seat belt use. These findings have implications for health communication practitioners who utilize social media sites as a means of promoting injury prevention. In future interventions that use Instagram to promote seat belt use, we recommend the use of rational appeals irrespective of the component of the post (photo, text, or caption) and use of fun photos with rational and positive emotional appeals rather than photos with fear or social appeals.

This study further confirmed that social media, especially Instagram, can be an effective tool to communicate the importance of using a seat belt to high school adolescents and young adults. In contrast with traditional mass media or institutional websites, Instagram allows users to generate their own content. For example, Instagram users in the *BuckleUp4Life* account generated creative photos with fun appeals such as a teddy bear or a dog wearing a seat belt. Future interventions can let adolescents and young adults create their own fun photos to promote seat belt use. Although ego and social appeals were not found to be attractive to the *BuckleUp4Life* Instagram account audience, we should not disregard the possible positive impact of ego and social appeals on audience members’ own Instagram account networks. Future interventions can recruit adolescents and young adults to post promotional messages on seat belt use, including seat belt wearing selfies, on Instagram. Interventions for other health topics on Instagram can also apply the different appeals that we found to promote health behaviors among adolescents and young adults.

### Limitations and Future Directions

Although this exploratory study was useful in providing insight into the usefulness of specific persuasive appeals, we only examined a limited number of posts (N=199). Furthermore, the sample size was not large enough to examine the limited number of comments besides likes on each post. Additionally, the *BuckleUp4Life* Instagram account was targeted toward high-school adolescents, some of whom were not drivers yet. However, as the web-based account was open to public, nursing students also responded to the posts, making it a mixed audience of high schoolers and nursing students who might have slightly different information processing preferences than high schoolers. Moreover, this account had a low engagement rate of 1.12% and the average interactions per post were 16 likes with 0 comments [22]. Thus, our findings may not be generalizable to a larger population. Future studies employing a large sample size should examine the content of the comments and explore the relationship between the different appeals and the content of the comments targeting adolescent or young adult groups specifically.

### Conclusion

This study is one of the first investigations to use a theoretical framework to evaluate how different persuasive appeals of an Instagram post influenced the responses of adolescents and young adults in a pilot seat belt promotion intervention. Our study demonstrates how practitioners should consider using specific persuasive appeals within specific post components when developing a public health intervention. Our findings suggest that in photos, adolescents and young adults prefer fun appeals, while in text and captions, rational and positive emotional appeals are most appealing. Rational appeal was more popular than ego and fear appeals, regardless of the location of the components (photos, text, and captions) in the posts. We recommend that public health practitioners use these results to inform the design and implementation of future public health road safety interventions through social media channels, especially Instagram.

## Abbreviations

ELM: elaboration likelihood model
RQ: research question
